# Coding the future: digital technologists and the constitution of the next system

**DOI:** 10.3389/fsoc.2025.1362848

**Published:** 2025-07-22

**Authors:** Dhruv Deepak, Ben Manski

**Affiliations:** Department of Sociology and Anthropology, Next System Studies, George Mason University, Fairfax and Arlington, VA, United States

**Keywords:** solidarity economy, next system, digital commoners, system change, constitutionalism, participatory design and governance, digital commonwealth, sovereignty

## Abstract

Digital technologists are coding the world of our immediate future. Digital commoners are a subset of digital technologists who aim to expand the spheres of life held in common, strengthen mutual aid, and create the conditions for shared participation in power. Relying on an understanding of technologists as activists, of technology as a movement, and of digital code as constitutional design, we analyze the digital commoners and their movement. Relying on a theory of the constitutive powers of digital technology in the areas of design, affordance, and sovereignty, we examine platform cooperatives, peer production systems, data sovereignty initiatives, and digital governance platforms, and analyze how these initiatives align with broader movements for system change. We argue that digital commoners are producing the elements of a digital commonwealth, a new form of democratic economic polity. Finally, we call on scholars and academic institutions to intervene and support digital commoning efforts, amplifying technologists’ capacity to code the future toward shared goals.

## 1 Introduction

Digital technologists are coding the world inhabited by the people of the 21st century. Among the internet-using majority, nearly half of waking hours are devoted to online media (Kemp, 2023). The formal global digital economy now comprises approximately 15% of gross world product (GWP) (Haar, 2023), with digitally-enabled platforms expected to generate 70% of new economic value over the 2020s (World Economic Forum, 2024). Nearly every subsystem of our world system—agricultural, artistic, ecological, educational, energy, governmental, health, legal, media, military, research, service, and transportation— is being reconstituted through digitalization.

The digital code for our world is not writing itself. At least, not as yet. Instead, rules determining much of our near future are being written by people with particular visions, goals, strategies, and understandings. This is a constitutional moment in which major technology corporations and state actors are primary drafters of a digitalized future. Our research, however, concerns a different set of constitutional authors: Technologists whose efforts aim to expand the spheres of life held in common, strengthen mutual aid, and create the conditions for shared participation in power. In short, they wish to grow the commons, build solidarity, and deepen democracy. These digital commoners are building systems for participatory planning, social ownership, and decentralized governance, coding a future where technology serves democracy and equity. Through platform cooperatives, participatory governance systems, and decentralized digital infrastructures, they are coding alternatives to centralized corporate control. We want to understand how their labor relates to the design of future society.

Our investigation of digital commoners emerges from our work with the Digital Commonwealth Project (DCP), an initiative of Next System Studies at George Mason University. Next System Studies investigate the relationships between systemic crises, system design, systemic movements, and the process of system change. As public sociologists engaged in Next System Studies and concerned with digital technology, we join other scholars of digital governance, social movements, constitutional change, and the political economy of technology in moving beyond observation to intervention, believing that academic institutions should work to bridge technical innovation and social transformation. This positionality and these commitments inform the framework elaborated here.

In the next part of this article, part 2, we introduce conceptualizations of technologists as activists, of technology as a form of movement, and of digital coding as a form of constitutionalism. Centering the agency of technologists and the historicity of technologization allows us to turn in parts 3 and 4 to a theorization of the constitutive powers of digital technology and of the practice of digital constitutionalism. Paying attention to the constitutive powers of design, affordance, and sovereignty, we argue, allows us to understand digital commoners as constitutional authors. In part 5, we analyze the efforts of digital commoners to create platform cooperatives, peer production systems, data sovereignty and digital rights initiatives, and digital governance platforms, each an important element for creating a digital commons. Then, with part 6, we describe how these, when linked with elements produced by the closely-related solidarity economy, municipalist, just transition, abolitionist, and democracy movements, enable the emergence of an alternative vision for reorganizing society that challenges both state-centric and corporate-driven technofuturist paradigms. As described in parts 8 and 9, we identify this alternative as a digital commonwealth, an economic political system where communities design digital technology to organize economic relations, embed democratic governance, and enhance collective wellbeing (Manski and Manski, 2018; Hanna et al., 2020). We conclude in part 10 with an argument for the necessity of thoughtful and purposive academic intervention. We believe that academic scholars and institutions should step into the work of digital commoning to help technologists integrate more effectively with the broader social movement toward shared goals.

## 2 Technologists as activists, technology as a movement, code as constitution

An activist is someone who has made the decision to take part in changing society; instead of simply living an ordinary life, the activist steps into the extraordinary work of making history (Flacks, 1988). A movement is collective action toward shared goals for changing society; when this action is directed toward a freer society for more people, it is described as a social movement (Touraine, 1988; Buechler, 2000). These definitions are drawn from the work of social movement scholars, and our approach to studying technological change draws significantly from social movement theory. For the purpose of our study, a technologist is a form of activist working for societal change, and technology itself is a form of movement.

Various scholars explain movements as responses to opportunities, threats, and other changes in the structure of society (McAdam et al., 2001; Tarrow, 2011) or as processes through which individuals come together on the basis of shared identities to engage in social conflict (Diani, 1992; Melucci, 1998; Flesher Fominaya, 2010). However, technologists are not merely responding to short-term changes in external conditions. Their work reflects a more strategic collective praxis aimed at reshaping societal systems (Taylor, 2000; Barker et al., 2013; Manski, 2019; Kioupkiolis, 2022). Rather than accepting a reductionist notion of technological determinism, which assumes that technology autonomously drives social and economic change (Bimber, 1990), we view technologists as strategic actors who are intentionally shaping technology to challenge entrenched power structures and reconfigure the world (Rozas et al., 2021; Scholz, 2023). While technology conditions the available pathways of movements, movements also shape the trajectory of technology (Mattoni, 2013; Flesher Fominaya and Gillan, 2017).

The decision to describe technologists as “a form of activist” relies on the recognition that there are different types of activists—student, youth, Indigenous, women’s, labor, LGBTQ+, among many others. All share in common their conscious agency engaged in the work of social change, yet each brings different knowledge, positional power, resources, and skillsets to their particular activism. However, just as not all students are activists, not all technologists consciously engage in the task of making history; what concerns us are the extraordinary actions of technologists who consciously produce technology in order to produce societal change, often embedding their visions and priorities into their creations (Scholz and Schneider, 2017).

Thomas Paine, the 18th century radical democrat, internationalist, and revolutionary, saw his design of the iron bridge as more than a feat of engineering; it was a political intervention aimed at strengthening the geographic and cultural unity of the nascent republic in the United States (Gray, 2016). Similarly, Marie Curie’s pioneering work in radioactivity was guided by humanist values, reflecting a commitment to improving the human condition and challenging prevailing social hierarchies (Zarevich, 2022). In contrast, Herman Hollerith’s mechanical tabulator, while a technical breakthrough, was designed to be used to categorize populations based on eugenic criteria, demonstrating how technology can reinforce harmful ideologies through seemingly neutral technical processes (Black, 2012; Zuberi and Bonilla-Silva, 2008).

These examples highlight the multiple valences of technological innovations and help clarify that technological change is, at least in part, produced ideologically. Technologists like Robert Owen, who integrated his political views into his experiments with community formation, pioneered social innovations that shaped labor rights and the cooperative movement (Podmore, 1905; Miliband, 1954; Gray, 2016). The rivalry between Nikola Tesla and Thomas Edison, often framed as the “War of Currents,” exemplifies how differing strategic commitments—radical versus incremental—can shape technological development (Bijker et al., 2012).

Note that the strategic commitments of technologists *as* technologists are toward particular visions of technology. This is what we mean when we write that “technology is a movement.” For technologists, technology in itself is a good and technological progress (however that is understood) is a primary goal. It must be said that the movement of technology is autonomous from other kinds of movements in society (e.g., social, national, religious, authoritarian) even as it relates to and overlaps with them. In this, we are in agreement with Charles Tilly that each kind of movement articulates an essentially distinct type of politics (Tilly and Wood, 2020). And we take from Eyerman and Jamison the understanding that movements are “actually constituted by the cognitive praxis that is entailed in the articulation of their historical projects,” and that, “the actual types of knowledge that a social movement articulates or is interested in obviously varies from movement to movement” (Eyerman and Jamison, 2007).

For social movements generally, and for the broad social movement of history, the movement’s cognitive praxis is oriented toward creating the conditions for universal human flourishing through the socialization of daily life (Touraine, 1981; Wright, 2010). To the extent that a movement is oriented toward the express articulation of system design, it is engaged in constitutionalism (Manski, 2020). Different movement forms may intersect. Thus, a movement may be religious and social; it may be national and authoritarian; it may be technological and constitutional. For us, therefore, digital constitutionalism is a practice around digital technology that is oriented toward next system design.

The history of the development of the internet epitomizes technology’s constitutional capacities. While now dominated by corporate interests, networked computing originated with J.C.R. Licklider’s vision of technology serving democratic exchange and collective intelligence (Pelkey et al., 2022). Licklider advocated for demilitarizing research and foresaw many of the key developments we now associate with the digital age, including cloud computing, artificial intelligence, and the political implications of networked computing (Dertouzos and Moses, 1979; Licklider, 1963). The displacement of his vision by an internet increasingly dominated by global corporations occurred in constitutional contests over technical standards, protocols, and governance structures. The battle to determine the internet’s technical standards and protocols was ultimately won by the Cisco, AT&T, IBM, Netscape, and Microsoft corporations (Pelkey et al., 2022; Monsees et al., 2023).

Today’s digital infrastructure signifies that constitutional victory, yet it also remains contested terrain where alternative constitutional visions persist (Wolfson, 2014). Indeed, digital technologists and activists continue to reclaim spaces for expression of their values and priorities (Mattoni, 2013). They are designing and implementing technologies that challenge the centralized control of information, allowing communities to assert authority over digital resources, and generating collective identity and solidarity among users and developers (Diani, 2000; Bauwens et al., 2019). In these cases, technologists often join with broad social movements. In particular, technologists are aligning with the long-building cooperative movement and with the much newer solidarity economy movement (Gordon Nembhard, 2021; Scholz, 2023; Papadimitropoulos and Malamidis, 2024). As we will describe in depth further in this article, coders are working with other activists to build alternatives to the extractive business models of corporate tech giants. Platform cooperatives, constructed as alternatives to platform capitalism (e.g., Amazon, Alphabet Inc., Uber), enable workers and users to collectively own and govern the digital platforms they rely on (Scholz, 2023). Building these new digital systems contributes not only to technological innovation but also to the democratization of social and economic relations, tending, we believe, to a new form of commonwealth (Manski and Manski, 2018; Bühler et al., 2023; Papadimitropoulos and Malamidis, 2024).

And that is the intent of these coders. They are deliberately writing the rules for a new society directly into the digital worlds they code. As such, they are constitutional authors. Where the 18th century saw the first triumphs of political constitutionalism, and the 19th and 20th centuries saw the emergence of economic and societal forms of constitutionalism, the 21st is experiencing the rise of digital constitutionalism (Teubner, 2010; Teubner and Golia, 2023). Here, the ordering and internal relations of an entire system is articulated in and enacted from programming protocols and code (De Gregorio and Radu, 2022).

## 3 The constitutive powers of digital technology

Digital technology is fundamentally reconfiguring the conditions of social life, from communication, law, and governance, to identity, exchange, and community. It possesses constitutive powers that define and organize the relationships between people, beings, and things. While these powers originate through the agency of technologists and historical movements, once enacted they exert their own autonomous force. As such, they warrant analysis. We identify three types of constitutive powers of digital technology: design, affordance, and sovereignty. Analyzing these powers illuminates how digital technology is reshaping the world and provides a framework for understanding its constitutional role. We believe that design, affordance, and sovereignty appear in all forms of constitutional action. However, as compared to classical constitutionalism, where there is usually a significant gap between formal and substantive law (which is to say, between the “law as text” and the “law in action”), the immediacy of digital code to material relations makes the empirical study of digital constitutionalism highly accessible.

Design is the deliberate articulation of relationships between people, beings, things, and systems. In digital contexts, design decisions determine who can access technology, how it can be used, and how power flows through it. Design channels and stabilizes possibilities while enabling new ones. It also reflects intentionality, which shifts the focus from merely regulating technology to using technology as a tool for reconfiguring social relations and creating new systems of governance.

When technologists design digital platforms and systems, they encode specific values, principles, and power relations into technical architectures (Winner, 1980; Dafermos and Söderberg, 2009; Pazaitis et al., 2022; Della Porta et al., 2022). This encoding process is explicitly constitutional; it establishes basic rules that shape interaction and institution formation.

The political significance of digital design can be seen well at scale: When transnational corporations like Google and Meta design their platforms, they create governance structures that, while formally private, perform traditionally public functions. These design choices constitute new forms of authority that often bypass traditional democratic processes (Zuboff, 2019; Birch, 2023). Conversely, the architecture of Wikipedia embodies principles of openness and collaboration. Its design allows anyone to contribute and edit, decentralizing knowledge production and challenging traditional hierarchies (Dafermos, 2020).

Affordance refers to the capabilities and actions enabled through a technology’s design. The concept originated in the work of perceptual psychologist Gibson (1986), but it was Donald Norman, a product designer and engineer, who popularized the notion of material “affordance” in the context of human-computer interaction (Norman, 2001). Affordances refer to how the internal structure of an object makes possible, and thus “affords,” particular uses. Technological affordances can be defined as a “type of action or a characteristic of actions that a technology enables through its design” (Earl and Kimport, 2011, p. 132).

The uses and products of a particular technology are conditioned by external forces, and thus, contingent. Yet emerging from their material structure, technologies possess their own autonomous power invoking specific uses and what they produce (Leonardi, 2012; Robey et al., 2012). The debate over affordance as a constitutive power is informative. One school of thought believes human agency is constrained by what people believe technology makes possible; another argues that the most crucial aspect of technology is how users put it into practice (Orlikowski, 2007). Still others remind us that technologies often possess affordances unplanned by their designers (Bimber, 1990). When technology and social structures interlock, a process of “imbrication” occurs, where the material agency of technology and the agency of human beings overlap (Leonardi, 2011). As technology mediates human action, it transforms social relations and materially alters the world (Cardullo et al., 2019).

Digital technology possesses affordances that mediate social relations and invoke particular uses, enabling new forms of agency (Earl and Kimport, 2011; Leonardi, 2012; Robey et al., 2012). For example, open-source platforms like GitHub provide affordances for collaborative software development, enabling technologists from around the world to contribute to shared projects (O’Neil et al., 2021). These affordances democratize access to technological tools, promoting innovation and collective problem-solving. But affordances can either constrain or enable democratic participation, depending on their design (Leonardi, 2011; Cardullo et al., 2019). Proprietary software and closed platforms limit user customization and lock communities into specific ecosystems, reducing their autonomy. The affordances of surveillance technologies, such as facial recognition systems, can reinforce authoritarian control and erode privacy rights, demonstrating the dual-edged nature of technological affordances (Cardullo et al., 2019).

Sovereignty refers to the receiving of general recognition of exclusive domain, and therefore, the related capacity to establish the rules for a particular field of action. In digital contexts, sovereignty is about who holds authority over digital infrastructures and how power is exercised. Sovereignty is often treated as a stable fact, yet there are always processes and movements underway that call sovereignty into question (Manski and Manski, 2018).

Historically, sovereignty was associated closely with different forms of state authority, and technological change was associated with changes in the organization of society and the form of the state (Marx, 2024). Thus, by the 18th century, the Western adoption of the printing press had enabled the spread of republican ideology and the rise of popular sovereignty (Young, 2006; Cameron, 2013). In the 19th century, steam engines and steel technology made possible the giant railroad corporations that secured for themselves a radically different relationship between corporate capital and the state (La Follette, 1897; Grossman and Adams, 1993). And in the 20th century, nuclear technology enabled a new geopolitical sovereign in the world system - the superpower - even as the AK-47 multiplied the insurgencies that undermined superpower hegemonies.

The development of digital technology in the 21st century is producing what appear to be contradictory forms of sovereignty. On the one hand, state and corporate surveillance capabilities have expanded dramatically, creating new challenges for collective economic rights and digital rights. The integration of artificial intelligence with surveillance systems enables unprecedented monitoring of social activity (Reich et al., 2021). Governments and corporations around the world have adopted advanced surveillance technologies to monitor and control populations, often under the guise of national security or public health.

Yet other innovations in digital technology have fragmented traditional forms of state sovereignty, enabling communities, workers, and networked users to assert authority over critical systems. Distributed ledger technologies such as Blockchain and Holochain introduce the possibility of decentralized governance, where authority is distributed across a network rather than concentrated in a single entity (Papadimitropoulos and Malamidis, 2024). Similarly, Indigenous data sovereignty movements assert control over digital knowledge systems, challenging colonial practices of data extraction and exploitation (Walter et al., 2021; Foxworth and Ellenwood, 2023).

## 4 The practice of digital constitutionalism

**Figure 1:**
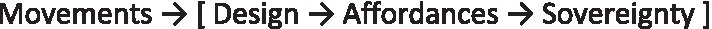
Primary order of operation for constitutionalization: movements and constitutive powers.

As we have noted, while written constitutions require independent and often elaborate enactment mechanisms, digital code narrows the gap between formal and substantive constitutionalism. When digital protocols specify how systems operate, those specifications immediately shape material behavior. This immediacy makes digital constitutional power both more precise and more totalizing than other constitutional forms. Technologists and their collaborators specify a system design, coders encode that design, new capabilities and actions are afforded by the new design, and new configurations of sovereignty follow from those affordances (Figure 1).

**Figure 2:**
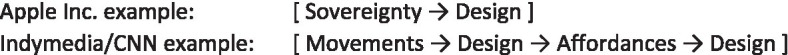
Alternative orders of operation for constitutionalization: movements and constitutive powers.

This is the primary order of operations in digital constitutionalism, but there are also other orders of operation. For instance, the exercise of sovereign power may facilitate particular kinds of new designs and limit others; consider the control that Apple Inc. exercises over its operating systems. Or consider a different order of operations: The present example of particular affordances may influence later design choices; once Indymedia.org demonstrated the power of DIY digital media tools, major media corporations like CNN emulated them with “citizen reporting” apps such as iReport (Daubs, 2016) (Figure 2).

We developed our theory of the constitutive powers of technology in order to explain the significance of the digital commoners and their movement. However, we believe that these same constitutive powers are also present in other recognized forms of constitutionalism, undertaken on municipal, national republican, regional/subnational, regional/continental/hemispheric, transnational/global, economic, societal, ecological, or other terrains. Historically, different sets of actors have wielded the constitutive powers of design, affordance, and sovereignty on these different terrains. Recent scholarship has shown that which actors engage in constitutional action on a particular terrain, and the degree to which they engage, significantly impacts outcomes. National constitutions initiated by social movements and drafted and ratified with authentic and inclusive popular participation tend to produce more democratic and equitable outcomes (Eisenstadt et al., 2017; Manski, 2020). While elite actors often dominate formal constitutional processes, transformative constitutional change requires the practical experiments and lived alternatives that movements generate. Digital constitutionalism thus depends on maintaining connection between technical constitutional authors and broader movements for social transformation (De Gregorio, 2022).

## 5 Digital commoners and their movement

Digital commoners are those who challenge the prevailing structure of the digital economy and seek to build alternative systems of technology governance and resource distribution. They are technologists, social movement activists, and community leaders who challenge the prevailing structures of the digital economy and seek to build alternative systems of technology governance and resource distribution. As such, they stand at the intersection of the movements of technology and of social progress. Unlike technologists whose labor falls within the ambit of established corporate or state institutions, digital commoners are committed to democratizing technology and placing it in the hands of communities (Scholz and Schneider, 2017; Dulong de Rosnay and Stalder, 2020). Their constitutional vision emphasizes collective ownership, democratic governance, and shared technological sovereignty. Through platform cooperatives, peer production systems, and community-controlled infrastructures, they write rules that aim to redistribute power and reshape economic relations (Bauwens et al., 2019; Walter et al., 2021; Gordon Nembhard, 2021; Bradford, 2023).

The movement of digital commoners encompasses a diverse array of actors and initiatives, united by their commitment to building open, distributed, and cooperative digital infrastructures. They draw from a broad spectrum of traditions, reflecting the multiplicity of approaches digital commoners employ to reimagine technology’s role in society (Scholz, 2023; O’Neil et al., 2021). They build on cooperative movement practices of collective ownership and democratic management; they incorporate solidarity economy principles of reciprocity and community benefit. This synthesis of approaches reflects their understanding that constitutional transformation requires both technical and social innovation.

One of the key projects of digital commoners is the creation of open and democratic digital infrastructures that challenge the centralized control of technology firms and governments (Hansen and Pang, 2023). The digital commons movement emerged from earlier experiments in technological democracy. During the 1990s, initiatives like Telestreet and Radio Mutiny demonstrated how alternative technical infrastructures could support social movements by enabling autonomous communication and coordination (Milan, 2016). Independent media projects built their own transmitters and networks, and Indymedia.org spawned hundreds of autonomous collectives around the world (Giraud, 2014; Milan, 2016; Stockwell and Manski, 2020). The early 2000s saw this experimentation expand through projects like Creative Commons, which developed new legal and technical frameworks for managing shared digital resources (Dobusch and Quack, 2008; da Rimini, 2010). Moreover, community-based internet initiatives serve as early examples of how digital commoners have sought to reclaim digital resources as public goods, aligning with the broader movement for technological sovereignty and the creation of civic, social, and digital rights (Cardullo et al., 2019).

Creative Commons helped to establish the digital commons as a legitimate space for cultural goods to be shared, used, and repurposed by communities worldwide (Dobusch and Quack, 2008; da Rimini, 2010). This symbolized a growing effort to balance human rights to access cultural goods with the need to protect and promote innovation, illustrating how digital commoners reshape the boundaries between public and private ownership in the digital realm (Dale and Kyle, 2016).

What distinguishes digital commoners as constitutional actors is their systematic engagement with technology’s constitutive powers. Their design practices deliberately encode democratic principles and cooperative relationships into technical architectures. They create affordances that enable collective governance and resource sharing. Through these efforts, they assert new forms of sovereignty that challenge both corporate and state control over digital systems.

The movement operates through multiple, overlapping initiatives that together constitute elements of an alternative economic system. Platform cooperatives establish democratic ownership over digital infrastructure. Peer production communities develop new models of collaborative value creation. Data sovereignty projects reclaim control over knowledge and information resources. Participatory governance platforms enable direct democratic decision-making. While diverse in their specific approaches, these projects share core constitutional commitments to expanding commons, strengthening mutual aid, and enabling shared participation in power.

Digital commoners operate in contested spaces where they must navigate the dual pressures of state regulation and corporate capture. For instance, the decentralized nature of community wireless networks like NYC Mesh and Freifunk in Berlin enables participants to bypass monopolistic internet service providers, asserting community sovereignty over digital infrastructure (O’Neil et al., 2021). However, such initiatives often face regulatory hurdles and limited access to resources, highlighting the structural challenges inherent in reclaiming the digital commons.

Similarly, the emergence of platform cooperatives like CoopCycle underscores the tension between collective ownership models and the profit-driven priorities of corporate platforms. CoopCycle’s worker-owned governance structure challenges the centralized control of gig economy platforms, demonstrating how digital commoners are redefining the rules of economic participation (Papadimitropoulos and Malamidis, 2024).

The 2008–2011 global protest wave marked a global surge of political resistance, with digital technologies at the center of these uprisings. Mass uprising such as the Green Wave in Iran, the Spanish Indignados, Arab Spring, Wisconsin, and Occupy used digital platforms to coordinate protests and engage in “connective action” (Treré et al., 2017). These highly networked movements relied on social media and digital communication tools to foster real-time coordination and mobilization. Platforms such as Twitter, Facebook, and teleconferencing technology became essential to organizing mass movements across borders, signaling how digital commoners leverage existing technological infrastructures to challenge state and corporate power (Milan, 2016).

For digital commoners, the primary means of struggle is innovation, and the main terrains they have contested until recently have involved those of regulation, sovereignty, and data:

- Design and governance: digital commoners seek to produce technology that is designed and governed by their intended users as commonly-owned property. This conflicts with state regulation, which often seeks to constrain, direct, or tax innovation, and with corporate interests, which tend to privatize quasi-public functions and extract value generated by users.
- Sovereignty: digital commoners prioritize individual, community, or shared sovereignty in the technologies they produce. This can conflict with both state sovereignty and corporate sovereignty.
- Data governance: digital commoners reimagine systems of ownership and decisionmaking around data, creating a more inclusive knowledge economy. They often work toward decentralized data systems to reduce power asymmetries, resist surveillance, and advocate for both transparency and privacy, thereby asserting data sovereignty.

Commoners seek holistic social institutions with distinct political economies and organizational dimensions, where the resources are digital in nature (Dulong de Rosnay and Stalder, 2020). They are building open, distributed, democratic, generative cyber-physical infrastructures that empower the public and expand technological sovereignty. Their worldview understands wealth creation as a social process based on community cooperation, not as an individualist process based on competition for scarce resources (Morris, 1992). Thus, digital commoners build on the experience of thousands of communities who have historically managed common resources successfully, including digital commons like Wikipedia and Linux (Wright, 2010; Torvalds and Diamond, 2002). Their movement is a proposal for a society where resources would be owned by everyone who needs to use them and managed in common by the users; the ultimate goals are democracy, equity, and solidarity.

Altogether, digital commoners come from different places and emphasize different values, but their labor has converged in the past decade as their visibility has increased. Their movement is not limited to isolated projects but represents a larger paradigm shift in how digital technology is conceived, developed, and governed. By embedding democratic principles into the design of their systems, enabling new forms of agency through affordances, and asserting sovereignty over digital infrastructures, digital commoners are constructing a new kind of economic and social order. They are consciously writing rules and enacting for the next system.

## 6 The constitutive powers of the digital commons

Digital commons initiatives demonstrate how technical design, affordances, and sovereignty can reshape social and political relations (Kostakis et al., 2015; Singh and Vipra, 2019). Through practical implementations of community-led technologies, from collaborative knowledge platforms to community-controlled networks and beyond, these projects develop foundational elements of alternative digital systems (Kioupkiolis, 2022).

One such alternative system, the digital commonwealth, represents a vision for reimagining society through technology—an integrated technological ecosystem where platforms and infrastructures enable new forms of social coordination (Manski and Manski, 2018; Hanna et al., 2020). This commonwealth transcends isolated resource management systems by creating interoperable digital architectures that facilitate fundamentally different social and economic relationships (Kioupkiolis, 2022). These relationships are built on shared governance, resource distribution systems, and common technological control.

We will return to the concept of the digital commonwealth in detail in parts 8 and 9. The sub-sections that immediately follow, however, examine four key types of digital commoning projects, analyzing how each mobilizes design, affordance, and sovereignty toward constitutional alternatives. Peer production systems establish frameworks for managing shared resources; platform cooperatives demonstrate new models of democratic enterprise; data sovereignty and digital rights initiatives develop technical frameworks for community control over information; and digital governance platforms enable new forms of collective decision-making.

In analyzing these implementations, we identify key approaches that together outline possible constitutional frameworks for a digital commonwealth. Each approach mobilizes technology’s constitutive powers in distinct ways. The technical architectures, interface capabilities, and governance protocols of these systems demonstrate how digital technologies can be structured to enable different patterns of resource access, participation, and control (Dulong de Rosnay and Stalder, 2020; O’Neil et al., 2021).

### 6.1 Peer production

Peer production represents one of the most developed experiments in digital constitutional innovation. It is the process by which individuals collaborate voluntarily to create shared resources, exemplifying how design, affordance, and sovereignty come together in digital systems (O’Neil et al., 2021).

Wikipedia’s design provides one of the most well-known examples of peer production. Its structure shows how digital systems can empower users to create, manage, and govern knowledge collectively, enabling large-scale collaborative production while maintaining democratic governance (Benkler, 2006; Hess and Ostrom, 2006).

Open technological standards and interoperable systems allow different initiatives to work together while maintaining autonomy. Peer production frameworks demonstrate how distributed collaboration can occur through shared protocols rather than centralized control (Bauwens et al., 2019).

- Wikipedia’s design embeds core principles of openness and decentralization into the platform architecture. Anyone with internet access can contribute content, while distributed moderation systems prevent centralized control over knowledge production (Dafermos, 2020).

Affordances in peer production systems enable collective governance and resource management. Through Wikipedia, contributors can create new pages, edit existing content, and engage in structured deliberation through discussion forums (Lanzi, 2023). These capabilities transform information consumers into active participants in knowledge creation, thereby creating a dynamic and responsive knowledge ecosystem. Various other projects demonstrate other kinds of affordances:

- Organizations like Creative Commons provide affordances that empower users to share and reuse cultural goods under flexible licensing agreements. This initiative illustrates how digital technology can be designed to enable collective agency and reshape the boundaries of intellectual property (Dobusch and Quack, 2008; da Rimini, 2010).
- As a contrasting example, the affordances provided by the Spatial Web indicate how digital technology can create novel opportunities for collaboration and resource sharing, by offering the tools to interact across both digital and physical spaces (Rene and Mapes, 2019). The Spatial Web attempts to code values such as data rights, biocentric design, and decentralized sovereignty into its protocols, reflecting the principles of a global commons across a new dimension of engaging with technology (*Ibid.*). Its founders see it as a more democratic and immersive alternative to the internet, integrating decentralizing technologies to merge cyber and physical worlds (Spatial Web Foundation, 2023).
- Freifunk in Berlin is an initiative where local residents have built decentralized, community-managed wireless networks using open-source technologies and community resources. Freifunk’s network enables residents to access the internet independently of commercial providers, democratizes control and management, and creates an atmosphere of shared ownership (Ridley-Duff and Bull, 2021).

New forms of sovereignty over information resources are evident with Wikipedia. Rather than concentrating control in either state or corporate institutions, the platform enables community self-governance through shared protocols and standards. Contributors collectively develop and enforce guidelines, creating a constitutional framework for managing shared digital resources (Han et al., 2023). This demonstrates how technical systems can enable democratic sovereignty over critical social infrastructure.

- Other projects, like Linux, demonstrate how peer production can create sophisticated technical systems through democratic coordination rather than hierarchical control (O’Neil et al., 2021).

### 6.2 Data sovereignty and digital rights

Data sovereignty initiatives demonstrate how communities can reclaim control over digital resources that shape their lives and livelihoods. These approaches illustrate how technical systems can either reinforce or transform power relations in the digital economy (Gray, 2023; Foxworth and Ellenwood, 2023).

Concerns about data colonization, sovereignty, and digital rights emerge across various communities and contexts. Worker organizations challenge the surveillance and algorithmic control enabled by platform labor management systems (Scholz, 2023). Privacy advocates develop tools and frameworks to protect personal data from commercial exploitation (Viljoen, 2021). Of particular interest are Indigenous communities that have been at the forefront of developing frameworks that ensure data practices align with community values and serve collective interests. This represents a global movement advancing constitutional innovations by reclaiming control over information about Indigenous peoples, lands, and cultural practices (Walter et al., 2021).

The design of community-controlled data systems explicitly incorporates principles of democratic governance and collective benefit. Technical frameworks require community participation in decisions about data collection, storage, and use. Access systems reflect shared values and priorities rather than commercial imperatives (Singh and Vipra, 2019). These design choices constitute practical mechanisms for exercising sovereignty over digital resources that increasingly shape economic and social life.

- Indigenous data governance frameworks embed principles of consent, self-determination, and cultural preservation into digital infrastructures (Bühler et al., 2023). These design choices constitute practical mechanisms for embedding principles of consent, cultural preservation, and community control into digital infrastructures (Gray, 2023; Bühler et al., 2023; Foxworth and Ellenwood, 2023).

The affordances of these systems enable communities to govern their data, protect cultural heritage, and ensure that digital practices benefit their members (Gray, 2023). Groups can establish collective protocols for data management, ensure information serves community interests, and prevent extraction or misuse of sensitive data (Pentland et al., 2021). These capabilities support broader movements for economic democracy and social solidarity by giving communities control over critical digital resources.

- In the city of Barcelona, an urban digital rights and data sovereignty agenda has been advanced since 2015 (Monge et al., 2022). The citizens of the city empowered themselves by deploying innovative policy and governance instruments to regain access and control over data.
- Indigenous communities, through their data sovereignty initiatives, can determine how information is collected, validate its accuracy, control its distribution, and ensure it serves community needs (Kukutai and Taylor, 2016). These technical capabilities support broader exercises of Indigenous sovereignty and self-determination (Carroll et al., 2020).

Sovereignty flows from these initiatives as communities exercise authority over the data infrastructures that increasingly mediate daily life, showing how technical systems can support rather than undermine collective self-determination and democratic governance (Bauwens et al., 2019). A key word in the previous sentence is “can.” We should keep in mind that they the assertion of data sovereignty can either reinforce existing power dynamics or be leveraged to democratize knowledge, access, and agency (Fung and Wright, 2001; Taylor, 2014).

- Community-owned initiatives like Zenzeleni in South Africa highlight the importance of sovereignty in digital infrastructures. By providing rural communities with locally controlled internet access, Zenzeleni empowers residents to bridge the digital divide while retaining wealth within their communities (R A et al., 2022).
- Indigenous data sovereignty empowers communities to reclaim control over their knowledge systems, positioning them as active agents in the digital realm rather than passive subjects of external governance (Gray, 2023).

### 6.3 Platform cooperatives

Emerging from the intersection of the solidarity economy, cooperative, and digital commoning movements, platform cooperativism has demonstrated positive outcomes for workers and communities by providing democratic alternatives to platform capitalism (Scholz, 2023). Successful platform cooperatives engage workers and communities in innovative models for ownership, profit-sharing, governance, decisionmaking and network-building (Saner et al., 2018; Graham et al., 2020).

At the 2023 Roots of Resilience conference in Kerala, India, technologists, activists, and community leaders from around the world convened to discuss how platform cooperatives and decentralized networks could provide a blueprint for equitable digital futures. This gathering represented a collective commitment to building digital infrastructures that prioritize social justice, climate resilience, and economic equity, reflecting the broader aspirations of the digital commonwealth (Platform Cooperativism Consortium and IT for Change, 2023a).

The design of platform cooperatives demonstrates how digital infrastructure can embed democratic ownership and governance into its fundamental architecture. CoopCycle, a worker-owned delivery platform, typifies this approach by redistributing control from corporate owners to the workers who use the system (Papadimitropoulos and Malamidis, 2024). Its design intentionally creates a system that promotes equitable distribution of value and collective decision-making, challenging the traditional models of corporate-controlled gig platforms, and embedding democratic decision-making processes into the platform’s core functionality.

- Platform cooperatives like CoopCycle and the Drivers Cooperative demonstrate how digital services can operate under worker and user control, ensuring value flows to those who generate it rather than external shareholders (Scholz, 2023; Papadimitropoulos and Malamidis, 2024).
- Namma Yatri is a direct-to-driver open mobility platform in Bangalore, India building a collective ecosystem of service providers on a common standard network, challenging the likes of Uber (Gurumurthy and Chami, 2020).
- Platform6 is a cooperative platform designed to provide mutual support to cooperative startups and social enterprises, offering technical resources, funding opportunities, and collaboration tools (Ridley-Duff and Bull, 2021).

The affordances of platform cooperatives enable workers to participate directly in operational governance. Unlike corporate platforms that restrict worker agency, cooperative systems provide tools for members to influence policies, control data, and determine value distribution (Scholz, 2023). These capabilities transform platform users from passive service providers into active participants in economic governance (Pazaitis et al., 2022; Kostakis et al., 2023).

- By asserting control over their labor and digital infrastructure, platform cooperatives like CoopCycle challenge the extractive models of gig economy platforms (Papadimitropoulos and Malamidis, 2024).

Distributed forms of sovereignty across platform cooperatives are evident in the growing global coordination conducted through initiatives like the Thiruvananthapuram Declaration. The Declaration’s vision extends beyond individual platforms to imagine interconnected systems that enable democratic economic coordination (Gurumurthy and Chami, 2020). Sovereignty is not limited to the digital realm but extends into economic and social systems, as they exercise control over the infrastructures that shape their lives (Calzati and Van Loenen, 2023).

- This gathering of technologists, activists, and community leaders outlined frameworks for building cooperative digital infrastructure that prioritize community control (Platform Cooperativism Consortium and IT for Change, 2023b).
- Solshare is a Bangladeshi data cooperative operating a decentralized energy trading platform. They provide clean, affordable energy to vulnerable communities, demonstrating how digital commons can intersect with sustainable development goals (Bühler et al., 2023).
- Unlike platforms like Uber or Deliveroo, where decisions are made by external shareholders, CoopCycle allows workers to govern the platform themselves, asserting digital sovereignty over their economic activities.

### 6.4 Digital governance

Digital governance initiatives demonstrate how communities develop technological systems for collective decision-making, policy formation, and resource allocation. These platforms and tools transcend traditional representative mechanisms by embedding participatory principles directly into their technical architecture. By creating accessible interfaces for citizen engagement, transparent processes for deliberation, and accountable mechanisms for implementation, these projects reconstitute relationships between communities and governance institutions, enabling direct democratic participation in decisions that affect daily life.

They work in specific ways: demonstrating commitments to knowledge democratization; instituting structural arrangements for horizontal decision-making and power redistribution; integrating multi-stakeholder participation; and emphasizing transparency to ensure their processes remain accessible to participants and the public.

Design choices in digital governance platforms have profound political implications. The Decidim platform, developed in Barcelona, places participatory democracy at the center of its architecture, enabling citizens to engage directly in governance processes. This reflects a commitment to inclusivity and transparency, demonstrating how design can challenge existing power structures and promote democratic values (Flanagan, 2022; Bynner et al., 2023). Some further examples illustrate this:

- Projects like Guifi.net in Spain showcase how design choices can decentralize control over digital infrastructure. Guifi.net is one of the largest commons-based wireless networks globally, enabling local autonomy and technological sovereignty by allowing communities to own and manage their networks (O’Neil et al., 2021).
- The proposed design of the publicly-owned British Digital Cooperative (BDC) suggests a federated structure that would establish local centers for technology development while maintaining democratic control through its workers and by citizen assemblies (Hind, 2019). It envisions public digital infrastructure that prioritizes privacy, security, and democratic deliberation over profit maximization.
- Community wireless networks like *NYC Mesh* and *Personal Telco Project* in Portland, offer decentralized alternatives to corporate internet service providers. These initiatives bypass the monopolistic control of traditional ISPs by allowing participants to build and manage their own networks, ensuring that internet access is a public good rather than a commodified service (O’Neil et al., 2021).

The affordances of these systems extend democratic participation beyond traditional representative mechanisms. With Decidim, citizens can propose policies, engage in structured deliberation, and participate in collective decision-making about public resources (Monge et al., 2022). These capabilities democratize governance processes that were previously restricted to elected officials or bureaucrats (Kostakis et al., 2015).

- Cooperatives like Som Energia show how digital platforms can enable community-driven approaches to resource allocation, bypassing traditional monopolistic structures (Giotitsas et al., 2015).
- Decidim’s success (and that of Barcelona’s technology democratization efforts), has inspired other similar initiatives around the world, demonstrating the potential for cultural change around technology (Flanagan, 2022; Bynner et al., 2023).

Sovereignty in digital governance manifests through multi-stakeholder structures that distribute authority across diverse participants. Organizations like the Open Mobility Foundation include both public members (cities, transit agencies, municipalities) with full decision-making power and non-public members (corporations, nonprofits, universities) who contribute to discussions and working groups (Hastings, 2024). This approach ensures that communities maintain control over digital systems while incorporating diverse expertise and perspectives.

- With Decidim, users exercise distributed sovereignty, asserting control over governance processes in a way that reflects principles of collective determination (Bynner et al., 2023).
- The proposed British Digital Cooperative’s “public option” would grant access to resources for civic engagement, cooperative economic development, and democratic deliberation - representing a radical reconfiguration of who controls digital infrastructure; sovereignty would be held by the public rather than private corporations (Ridley-Duff and Bull, 2021).
- Fairbnb.coop, a cooperative platform that challenges the centralized control of traditional sharing economy platforms like Airbnb, offers a socially responsible alternative that reinvests profits into local communities and is governed democratically by its users and hosts (Papadimitropoulos and Malamidis, 2024). It demonstrates the reclaiming of sovereignty over platforms, supporting local economies and social goals such as producing democracy, over profits (Kostakis et al., 2023; Falanga, 2024).

## 7 The solidarity economy, community wealth, and the digital commons

These examples of digital systems, where resources are owned, managed, and governed across multiple institutions and locations by their communities, show us how digital architecture can be designed to serve community needs rather than extract value. Som Energia’s energy management platform, for instance, integrates cooperative principles directly into its technical protocols, enabling members to collectively govern their resources. Other examples like The Drivers Cooperative use similar principles to build alternative ride-sharing applications that distribute earnings equitably. Such implementations prove that alternative ownership models can be effectively encoded into functional digital systems at scale.

Notably, digital commoners’ projects intersect with a variety of contemporary community-based economic development strategies. Community Wealth Building (CWB) is an economic development strategy that focuses on transforming local economies through community control and direct ownership of assets (Guinan and O’Neill, 2020). CWB aims to address wealth inequality by challenging traditional economic development models, pursuing cooperatives and other democratic enterprises that distribute economic power more equitably (*Ibid*). In some regions, CWB projects have developed complementary digital infrastructure that strengthens local economic ecosystems through procurement platforms that connect anchor institutions with local businesses, digital marketplaces for cooperative enterprises, and data visualization tools that track community ownership and wealth retention.

Shared possibilities are also reflected in the solidarity economy (SE), an socio-economic system designed to improve the quality of life for the group or community on the basis of solidarity, sometimes through collective enterprise or community-based giving (Gordon Nembhard, 2021; Fraisse, 2013). SE principles feature democratic governance, inclusive participation, political empowerment, and struggles for social justice (Gordon Nembhard, 2021).

Where the SE movement has focused on advancing labor rights out of concern for the future of work, it has yielded substantial socio-technological innovation (Borzaga et al., 2019; Graham et al., 2020; Della Porta et al., 2022). For example, in Brazil, solidarity economy networks have created incubation platforms for technological solutions, producing software specifically designed for cooperative management, mutual aid coordination, and collective resource allocation (Dubeux, 2013). Similar implementations exist across Latin America, Europe, and parts of Asia, where digital marketplaces, resource-sharing applications, and cooperative financing platforms operate as practical alternatives to corporate-controlled digital systems.

The solidarity economy has developed a substantial ecosystem of digital platforms with significant, yet largely unrealized potential for integration with digital commons projects. The US Federation of Worker Cooperatives maintains management information systems specifically designed for worker-owned businesses with over 1,300 cooperatives and 15,000 workers using these platforms. Democracy at Work has built media production and distribution infrastructure for cooperative economic education, making technical knowledge more accessible across sectors. The Tech Workers Coalition has developed communication tools that enable organizing within the technology industry itself, connecting highly compensated tech workers with gig economy laborers in shared campaigns. Each of these examples represents a technical implementation of solidarity principles, not merely aspirational values.

These technical systems remain largely disconnected from digital commons initiatives despite their clear complementarity. For instance, the International Cooperative Alliance’s coordination platforms, representing over one billion cooperative members globally, but this operates separately from the Global Tapestry of Alternatives’ networking infrastructure, which links social movements across geographic and sectoral boundaries. And the US Solidarity Economy Network has created mapping platforms that document alternative economic initiatives across multiple sectors, also functioning independently.

Despite parallel developments, engagement between digital commoners and SE or CWB practitioners remains sporadic rather than systematic, reflecting a broader challenge. While successful examples of integration exist — such as the Platform Cooperativism Consortium and IT For Change’s joint Roots of Resilience conference (Platform Cooperativism Consortium and IT for Change, 2023b) — these represent temporary convergences rather than sustained coordination. The separate governance structures of these movements, each with their own participatory processes and decisionmaking protocols, create significant barriers to ongoing collaboration. Additionally, variations in motivating principles and strategic priorities often result in misalignment even when technical compatibility exists.

Together, the four approaches presented earlier (in parts 6.1 through 6.4) demonstrate how digital commoners mobilize technology’s constitutive powers toward democratic ends. A broader, uniting constitutional vision is missing, however, as is connective digital infrastructure. For this, the digital commonwealth offers a framework for integration; by providing coordination mechanisms for aligning priorities and resources, platforms from different traditions could become interoperable components of a larger ecosystem. The techniques, protocols, and governance models developed in various contexts could strengthen one another through deliberate integration, creating more robust alternatives to dominant digital systems. However, we shall soon see, significant barriers threaten this potential integration as well as the continued development of digital commons projects themselves.

### 7.1 Emerging threats to the digital commons

The digital infrastructure created by commoning projects faces mounting challenges that threaten to undermine its continued development and effectiveness. These challenges directly target the constitutional elements we have examined—compromising carefully crafted designs, restricting democratic affordances, and undermining community sovereignty over digital resources. The preceding analysis of digital commons technologies and their intersections with solidarity economy and community wealth systems reveals particularly vulnerable points where external pressures could significantly compromise these alternative digital architectures. These challenges emerge from multiple directions, requiring coordinated responses that combine technical innovation with social mobilization.

#### 7.1.1 Political realignment and corporate capture

Recent political developments in the United States following the 2024 election present a significant threat to alternative digital infrastructures. While the technology sector has historically maintained some independence from government influence, in recent months we have witnessed a major realignment of the relationship between major technology corporations and the White House. The transformation of the U.S. Digital Service into the “Department of Government Efficiency” exemplifies this shift, replacing public-interest technology governance with market-driven efficiency metrics. This institutional restructuring directly threatens the regulatory frameworks that digital commons rely upon for protection against corporate encroachment.

The physical presence of technology billionaires like Elon Musk in White House operations enables the direct implementation of technological visions fundamentally opposed to commons-based principles. These corporate executives can now encode their priorities—surveillance capitalism, data extraction, and algorithmic control—directly into federal technology policy. The broader pattern of billionaire appointments to Cabinet positions signals a governance approach that prioritizes private sector technological solutions over democratic alternatives. Cooperative platforms, community-controlled data systems, and peer production infrastructures face not only market competition from better-resourced platform capital but also unfavorable regulatory changes designed to privilege proprietary systems over commons-based approaches.

#### 7.1.2 Technological disruption and generative AI

The rapid development of generative artificial intelligence systems poses a critical challenge to digital commoners in the form of technological disruption emerging in spaces that democratic governance has yet to anticipate. When OpenAI released ChatGPT in November 2022, it demonstrated how quickly proprietary AI systems could redraw the boundaries of technological possibility without public oversight or democratic input (Future of Life Institute, 2023). Generative AI accelerates technological innovation but often does so in ways that exacerbate inequalities and concentrate power, while also raising concerns about accountability and the ethical use of technology (Morozov, 2023; Bradford, 2023). Such developments reinvigorate fundamental debates about who controls and benefits from technology.

For digital commoners, the rise of AI poses multiple challenges. First, it disrupts existing social and economic relations, creating new asymmetries of power. Second, the computational resources required to develop and deploy comparable AI systems often exceed what community-governed projects can access, creating an expanding capability gap between corporate and commons-based digital infrastructure. Third, these systems disrupt existing digital infrastructures by automating functions previously performed through collective human effort. Peer production systems like Wikipedia, which rely on distributed human knowledge contribution, face potential obsolescence as proprietary AI systems can generate similar content (and are built on existing internet content) with minimal human involvement.

The technical architecture of these AI systems typically embeds values contrary to digital commons principles—centralized control, opaque operation, and data extraction—making them difficult to adapt for democratic governance. Addressing these challenges will require strategic collaboration and innovation, as well as advocacy for regulatory frameworks that prioritize public accountability and transparency (Morozov, 2023; Bradford, 2023).

#### 7.1.3 Corporate encroachment on digital sovereignty

The dominance of a few technology corporations over digital infrastructures continues to undermine the principles of openness and decentralization that digital commoners champion. From the monopolization of digital platforms to the commodification of data, these corporations increasingly control the digital economy, extracting value without contributing to community wellbeing (Zuboff, 2019; Birch, 2023). Moreover, they often appropriate innovations originally developed by commons-based initiatives. Platform cooperatives and community-controlled networks, for example, face increasing pressure from corporate competitors with vastly greater resources (Scholz, 2023). This process of enclosure threatens to undermine the foundational premise of digital commoning, which is that technological resources can and should serve common rather than private interests.

#### 7.1.4 Expanded surveillance infrastructure

Digital surveillance technologies represent a growing threat to the technical viability of commons-based systems. The integration of artificial intelligence with commercial and governmental monitoring capabilities has created unprecedented technical means for tracking, analyzing, and controlling digital activity (Reich et al., 2021). These surveillance systems directly undermine core technical requirements of digital commons platforms: user privacy, secure communication, and community autonomy.

State and corporate surveillance capabilities have expanded dramatically, creating new challenges for collective economic rights and digital rights. Governments around the world have adopted advanced surveillance technologies to monitor and control populations, often under the guise of national security or public health. These practices undermine digital rights, including privacy, freedom of expression, and access to information (Sekalala et al., 2020; Afriat et al., 2021).

The technical infrastructure of surveillance operates across multiple layers—network monitoring, device access, data collection, and behavioral analysis—creating comprehensive visibility into digital activities. These systems can identify users of alternative digital platforms, monitor their communications, and potentially disrupt their operations. For community-controlled networks and cooperative platforms, this surveillance infrastructure presents a spectral challenge to their technical security and operational independence.

Open-source technologies and community-controlled data systems require secure communication channels to function effectively. Yet as surveillance capabilities expand through both technical advancement and regulatory permission, maintaining secure channels becomes increasingly difficult. Digital commons infrastructure must continuously evolve defensive capabilities just to maintain basic operational security, diverting resources from more productive development efforts (Viljoen, 2021).

#### 7.1.5 Fragmentation of the digital commons

The digital commons itself shows signs of fragmentation as different initiatives pursue divergent technical and organizational approaches, threatening its long-term viability. While particular projects have developed sophisticated platforms for specific purposes, the technical integration necessary for a coherent alternative to dominant systems remains underdeveloped. Peer production platforms, cooperative management systems, and participatory governance tools often use incompatible technical standards, authentication systems, and data structures. These technical incompatibilities reflect both the decentralized development of these systems and the absence of coordinating infrastructure.

Projects can struggle to scale beyond local experiments to challenge dominant corporate platforms (Dulong de Rosnay and Stalder, 2020). While digital commoners are united by shared principles, the movement remains highly decentralized. A lack of coordination can result in duplication of efforts, inefficiencies, and missed opportunities for collaboration, undermining the impacts of a coherent constitutional vision (O’Neil et al., 2021).

The lack of technical integration mechanisms prevents these systems from functioning as a coherent ecosystem. This technical fragmentation makes individual platforms more vulnerable to external threats, limits their ability to scale beyond niche applications, and reduces their collective impact as alternatives to dominant digital systems.

Something more is needed. Digital commoners have produced various elements of a possible next system. Yet those elements, as promising and potentially powerful as they are, face mounting challenges. A superstructure could enable more effective integration and resilience.

## 8 The emergence of a digital commonwealth

Various kinds of territories, polities, and communities have gone under the name “commonwealth,” with many uses of the term suggesting a self-governing community that shares responsibility for the general welfare of the people of a somewhat large and diverse territory. Recent renditions of the concept of “commonwealth” center on shared resources and collective ownership, emphasizing democratic participation, distributed power, and egalitarianism, as opposed to private or state control of capital (Linebaugh, 2008; Hardt and Negri, 2011; Federici and Linebaugh, 2019).

Historical examples of commonwealth projects like Eugene V. Debs’ American Cooperative Commonwealth and Canada’s Cooperative Commonwealth Federation demonstrated how economic institutions could be designed to shape political relations (Brommel, 1971; Deshaies, 2019). What these older conceptions of commonwealth have in common is that they invert state-centered political economy into a participatory economic politics in which the goal is the construction of a particular type of democratic economic polity (Winch, 1977; Alperowitz, 2017).

We borrow from both recent and historical commonwealth concepts to define a digital commonwealth as a holistic system where digital technology is designed to organize economic and social relations that prioritize democracy, equity, sustainability, and solidarity. Thus, the digital commonwealth is not simply a rebranding of the digital commons; rather, it is an extension and integration of various cooperative, commons-based, and participatory efforts into a new form of economic polity (Manski and Manski, 2018). Building upon the various initiatives of the digital commons, which often operate in domain-specific silos, the DC synthesizes these projects into a coherent program for restructuring digital governance, ownership, and life (Hanna et al., 2020). Where existing commons projects often focus on protecting particular sets of resources, commonwealth-building seeks to establish new frameworks for economic organization, across different scales and domains, while maintaining democratic participation (Restakis, 2021; Kioupkiolis, 2022).

## 9 Elements of a digital commonwealth

The digital commonwealth provides a logical next step in the work of digital commoning. Digital commoner initiatives demonstrate how technical systems can embed democratic principles into the infrastructure of daily life. Platform cooperatives establish collective ownership over digital services. Peer production systems enable collaborative resource management; open-source technologies and knowledge commons are a reservoir for innovation. Data sovereignty movements assert community control, enabling communities to manage digital resources according to shared values. Participatory governance platforms facilitate direct democratic decisionmaking and engage communities in shaping technological development. Thus, many if not most of the constituent elements of a digital commonwealth are already present:

1. Democratic ownership and governance of digital platforms is widely practiced in collective decisionmaking about technological development and deployment (Scholz, 2023; Papadimitropoulos and Malamidis, 2024).
2. Data sovereignty and community control over data enable ordinary people to manage digital resources according to shared values and priorities, to prevent extractive data practices, to safeguard privacy from surveillance, and to collaborate in economic production (Singh and Vipra, 2019; Walter et al., 2021).
3. Open technological standards and interoperable systems allow different initiatives to work together while maintaining autonomy; this promotes collaborative innovation and resists against private enclosure of shared resources (Bauwens, Kostakis).
4. Participatory design and governance frameworks facilitate democratic decisionmaking across different scales, combining local autonomy with broader coordination, allowing communities to address shared challenges while maintaining control over local resources (Flanagan, 2022).
5. Solidarity economies and community wealth building center the equitable distribution of value, ensuring that resource allocation recirculates value in local economies, and protecting against extractive practices (Giotitsas et al., 2015).
6. Ecological design principles center sustainability, creating the conditions for a socioeconomic system that lives within the ecological capacities of the biosphere (Kostakis et al., 2023; Smith, 2024).

### 9.1 Building the economic polity of the digital commonwealth

The digital commonwealth represents more than a collection of democratic and solidaristic technology projects, it signifies an emerging economic polity with distinct institutional forms and organizational logics. As we have written, where classical political economy is more often concerned with how the design of political institutions shapes economic behavior, the digital commonwealth inverts this relationship, creating economic institutions that enable new forms of democratic participation. This inversion occurs through the deliberate design of technology that embeds democratic principles in economic infrastructure. Platform cooperatives do not simply provide alternative ownership structures, they establish new frameworks for economic coordination that prioritize collective benefit over private accumulation (Scholz, 2023). Community-controlled data systems do not just protect privacy, they enable new forms of participatory planning and resource allocation (Pentland et al., 2021).

And it is here that we begin to see a series of formative processes through which various elements of the digital commons synthesize into the systemic alternative of the digital commonwealth. First, democratic ownership of digital platforms creates new circuits of value that keep resources circulating within communities rather than being extracted by external capital (Papadimitropoulos and Malamidis, 2024). Second, peer production systems establish frameworks for managing shared resources that transcend both market and state logics (Benkler, 2006; O’Neil et al., 2021). Third, participatory governance enables direct democratic control over economic decision-making. Fourth, open technological standards promote autonomy, innovations, and protection from private enclosure (Bauwens et al., 2019). Finally, ecological principles become embedded in economic organization through technology that the biosphere and biological systems as a global commons, and not an externality to be managed (Giotitsas et al., 2015).

The elements of the digital commonwealth gain transformative power through their interaction and mutual reinforcement. While individual projects demonstrate important innovations, their true potential emerges from the synergies between different approaches to democratic technology governance (Dafermos, 2017; Bauwens et al., 2019). When combined, these elements create a robust digital ecosystem where governance, resource allocation, and technological innovation are participatory, equitable, and sustainable.

A thriving digital commonwealth enhances social relations and wellbeing by providing a common technological backbone for collaboration, co-production, and equitable resource allocation. Digital commoners act as key protagonists in this ecosystem, mobilizing the constitutive powers of digital technology—design, affordance, and sovereignty—to champion values like openness, inclusivity, cooperation, and equitable resource distribution (Akerlof et al., 2023). Together, these processes constitute a distinctive economic logic that represents a fundamental departure from both platform capitalism and state-centric digital authoritarianism (Singh and Vipra, 2019; Jung, 2022).

### 9.2 Synergies and future potential

Imagine a future where governance systems, platform architectures, and digital infrastructures are all interoperable and aligned with commons-based principles. In this system, users could seamlessly interact across various platforms, engaging in participatory decision-making processes that prioritize technological sovereignty, privacy, and community wellbeing. Consider how platform cooperatives and data sovereignty initiatives complement each other. Worker-owned platforms like CoopCycle establish democratic control over economic infrastructure, while data sovereignty frameworks ensure the information generated through platform activity serves collective interests. Together, they create systems where both physical and digital resources remain under community control (Scholz, 2023; Walter et al., 2021). Additionally, the interoperability of platform cooperatives with participatory democracy platforms like Decidim could create a seamless ecosystem for civic engagement and economic empowerment.

Similarly, peer production systems become more powerful when combined with participatory governance platforms. Wikipedia’s content creation model demonstrates collective knowledge production, while systems like CitizenLab provide frameworks for democratic decision-making. Their integration suggests possibilities for large-scale coordination that maintains both efficiency and democratic participation (O’Neil et al., 2021; Flanagan, 2022).

The potential for systemic transformation becomes visible when examining successful combinations of these elements (Monge et al., 2022). Several promising developments suggest expanding potential for the digital commonwealth. First, growing coordination between different initiatives creates possibilities for broader systemic change. The Platform Cooperativism Consortium demonstrates how networks of democratic technology projects can share resources and strategies while maintaining local autonomy (Platform Cooperativism Consortium and IT for Change, 2023a). Second, new technical capabilities enable more sophisticated forms of democratic coordination. Advances in cryptographic protocols, for example, create opportunities for secure, transparent governance at larger scales (Kostakis et al., 2023). Finally, growing recognition of platform capitalism’s limitations creates openings for alternative models. As concerns about surveillance, labor exploitation, and ecological damage mount, democratic alternatives gain credibility (Zuboff, 2019; Reich et al., 2021).

Integrating the efforts of digital commoners with broader social movements and leveraging the constitutive powers of digital technology, the digital commonwealth offers a vision of an alternative system. However, as discussed in parts 7.1 through 7.1.5, realizing this potential requires deliberate effort to address challenges posed by fragmentation, resource scarcity, and concentrated corporate power and state power.

## 10 Conclusion: a next system and the necessity of intervention

At the intersection of technology and the movement for social progress, burns a question: Who is present at the design of technological protocols? Who is in the room when the constitution of the next world is written?

Technologists can be understood as coders of the future. Their movements produce technologies imbued with constitutive powers. Their labor should be understood as a form of constitutionalism. They embed their designs for the next world into digital code. Their technology is all around us and in us. It is technology that we increasingly rely upon, and that organizes much of economic, social, and political life.

Digital commoners share with other technologists a belief in technological progress. Attention to this belief reminds us that *technology is itself a movement,* one that has had a complex and oftentimes contradictory relationship to the broad social movement of history. Digital commoners operate near the intersection of the movements for technological and social progress. Yet their experiences, worldviews, and knowledges are limited, and they are less resourced than the digital technologists of global capitalism. They are unlikely to achieve their goals on their own.

Digital commoners share with other members of the broader social movement a commitment to democratic transformation, yet significant gaps often separate technical innovation from social movement building. Movements for social progress can benefit immensely from the technical expertise, innovation, and vision of technologists. Conversely, digital commoners require the organizational strength, community networks, and political strategies that other kinds of social movement activists bring to the table. This divide limits both groups’ transformative potential (Scholz and Schneider, 2017; Platform Cooperativism Consortium and IT for Change, 2023a).

### 10.1 The role of academia in shaping the digital commonwealth

Academic institutions have unique capabilities and responsibilities in supporting digital constitutional innovation. They are uniquely positioned to act as conveners, bridging the gap between digital technologists and social movements. Research partnerships help illuminate technology’s constitutive powers while identifying strategic opportunities for intervention. Educational programs that combine technical training with social analysis prepare new generations of constitutional technologists. Institutional resources can support experiments in democratic technology development, helping bridge the gap between technical possibility and social transformation. Through research, education, and public engagement, scholars can:

1. Facilitate collaboration by creating spaces where technologists, activists, policymakers, and community members can come together to co-design solutions for the digital future.
2. Offer critical perspective by analyzing the implications of digital technology, identifying both opportunities and risks for democracy, equity, solidarity, and sustainability.
3. Provide institutional support by developing programs that help technologists, entrepreneurs, scholars, and other workers align their skills and careers with the values of the digital commonwealth, and support ongoing projects that embody its principles.

For instance, the Digital Commonwealth Project (DCP) of Next System Studies at George Mason University takes inspiration from Touraine (1983) method of sociological intervention, working with community, technology, academic, and movement partners to democratize economic and political systems through digital technology. The DCP’s interventions have included a partnership with NOVA Web Development Cooperative to implement applications of Decidim by Democratizing NOVA (a university-community partnership building capacity for economic and other forms of democratization in Northern Virginia), by the Next Constitutions Research Lab, and by the Next System Teach-Ins process. The DCP has also launched Computing for the Common Good, a school-to-college-to-workforce pathway to develop a stable regional workforce skilled in participatory digital technology.

As multiple types of next system alternatives emerge in response to accelerating crises, the digital commonwealth offers distinctive promise. The convergence of digital commoners with other currents of social transformation creates opportunities for fundamental change. Academic institutions can help strengthen these connections while maintaining focus on democratic principles. This intervention serves not just digital commoners but also the broader project of creating technology that enables rather than constrains human flourishing.

### 10.2 Toward a digital commonwealth

As the elements of a digital commonwealth emerge on many terrains in many countries, the possibility of a new economic polity becomes visible: A next system with its own logics, powers, and understandings of personhood, sovereignty, community, and solidarity. The work of digital commoners is beginning to converge with solidarity economy, community wealth building, local exchange, participatory planning, municipalist, just transition, constitutional reform, and other initiatives. This convergence is necessary to create a robust and resilient digital commonwealth that can challenge the dominant paradigms of digital capitalism and state control (Jung, 2022). However, it is also insufficient. The realization of a digital commonwealth requires a multifaceted approach that addresses not only technological and economic aspects but also social, cultural, and political dimensions. It necessitates the development of new legal frameworks, educational initiatives, and cultural shifts that can support and sustain these alternative models of digital governance and economic organization (Singh and Vipra, 2019; Pentland et al., 2021; Viljoen, 2021). Moreover, it requires ongoing efforts to bridge digital divides, ensure equitable access to digital resources, and foster digital literacy across diverse communities.

If as we have argued, digital design is a constitutional process, then the construction and consolidation of a digital commonwealth that presents a credible next system alternative will require popular participation. We know that constitutions work best for the most people when the process of constitution-making is truly participatory, inclusive, and taken at the initiative of a social movement (Eisenstadt et al., 2017; Manski, 2018). Just as constitutions derive their legitimacy from the involvement of ordinary people, a digital commonwealth will only succeed if it is shaped by the diverse communities it aims to serve. This necessitates ongoing engagement with the public, the development of new legal and institutional frameworks, and a commitment to equity and inclusivity.

Similarly, popular movements for social progress need closer partnerships with digital commoners. They need the technical expertise of coders and the capital of the technology sector. Beyond this, they require better understandings of the implications of technological change. Just as coop members and community activists have special knowledge often missing to technologists, so too do technologists possess special forms of knowledge needed by communities.

In this period of accelerating system failures brought about through technological change, deepening austerity and social inequality, and ecological collapse, the work of digital commoners offers a hopeful alternative to the extractive and exploitative models of much of the digital economy. But their success requires sustained collaboration with others, and academic institutions have a duty to facilitate, analyze, and support this collaboration. The constitution of a digitalized 21st century is being written, and we must all become its authors.

## Data Availability

The original contributions presented in the study are included in the article/supplementary material, further inquiries can be directed to the corresponding authors.

## References

[ref1] Afriat H. Dvir-Gvirsman S. Tsuriel K. Ivan L. (2021). “This is capitalism. It is not illegal”: users’ attitudes toward institutional privacy following the Cambridge Analytica scandal. Inf. Soc. 37, 115–127. doi: 10.1080/01972243.2020.1870596

[ref2] Akerlof K. L. Timm K. M. F. Chase A. Cloyd E. T. Heath E. McGhghy B. A.. (2023). What does equitable co-production entail? Three perspectives. Commun. Sci. 2:e2022CSJ000021. doi: 10.1029/2022CSJ000021, PMID: 39712630

[ref3] Alperowitz G. (2017). “Principles of a pluralist commonwealth: Introduction,” in *Principles of a Pluralist Commonwealth*. The Democracy Collaborative.

[ref4] Barker C. Cox L. Krinsky J. Nilsen A. G. (2013). Marxism and social movements. Leiden: BRILL.

[ref5] Bauwens M. Kostakis V. Pazaitis A. (2019). Peer to peer: The commons manifesto. London: University of Westminster Press.

[ref6] Benkler Y. (2006). The wealth of networks: How social production transforms markets and freedom. New Haven, CT: Yale University Press.

[ref7] Bijker W. E. Hughes T. P. Pinch T. J. (2012). The social construction of technological systems: New directions in the sociology and history of technology. London: MIT Press.

[ref8] Bimber B. (1990). Karl Marx and the three faces of technological determinism. Soc. Stud. Sci. 20, 333–351. doi: 10.1177/030631290020002006

[ref9] Birch K. (2023). There are no markets anymore: from neoliberalism to big tech (state of power 2023). Amsterdam: Transnational Institute.

[ref10] Black E. (2012). War against the weak: Eugenics and America’s campaign to create a master race. Highland Park, MI: Dialog Press.

[ref11] Borzaga C. Salvatori G. Bodini R. (2019). Social and solidarity economy and the future of work. J. Entr. Innov. Emerg. Econ. 5, 37–57. doi: 10.1177/2393957518815300

[ref12] Bradford A. (2023). Digital empires: The global Battle to regulate technology. 1st Edn. New York, NY: Oxford University Press.

[ref13] Brommel B. J. (1971). DEBS’S cooperative commonwealth plan for workers. Labor History 12, 560–569. doi: 10.1080/00236567108584180

[ref14] Buechler S. M. (2000). Social movements in advanced capitalism: The political economy and cultural construction of social activism. Oxford: Oxford University Press.

[ref15] Bühler M. M. Calzada I. Cane I. Jelinek T. Kapoor A. Mannan M.. (2023). Unlocking the power of digital commons: data cooperatives as a pathway for data sovereign, innovative and equitable digital communities. Digital 3, 146–171. doi: 10.3390/digital3030011, PMID: 40278680

[ref16] Bynner C. Escobar O. Weakley S. (2023). Facilitators as culture change workers: advancing public participation and deliberation in local governance. Local Gov. Stud. 49, 738–758. doi: 10.1080/03003930.2023.2190586

[ref17] Calzati S. Van Loenen B. (2023). A fourth way to the digital transformation: the data republic as a fair data ecosystem. Data Policy 5:e21. doi: 10.1017/dap.2023.18

[ref18] Cameron M. (2013). Strong constitutions: Social-cognitive origins of the separation of powers. New York, NY: Oxford University Press.

[ref19] Cardullo P. Di Feliciantonio C. Kitchin R. (2019). The right to the Smart City. England: Emerald Publishing Limited.

[ref20] Carroll S. R. Garba I. Figueroa-Rodríguez O. L. Holbrook J. Lovett R. Materechera S.. (2020). The CARE principles for indigenous data governance. Data Sci. J. 19:43. doi: 10.5334/dsj-2020-043

[ref21] da Rimini F. (2010). “Social technologies and the digital commons” in Handbook of research on social interaction technologies and collaboration software. eds. Dumova T. Fiordo R. (New York, NY: IGI Global), 601–622.

[ref22] Dafermos G. (2017). *The Catalan integral cooperative: an organizational study of a post- capitalist cooperative* [organizational study] P2P foundation. New York, NY: Robin Hood Coop.

[ref23] Dafermos G. (2020). “Prophets and advocates of peer production” in The handbook of peer production. eds. O’Neil M. Pentzold C. Toupin S.. 1st ed (New York, NY: Wiley), 87–100.

[ref24] Dafermos G. Söderberg J. (2009). The hacker movement as a continuation of labour struggle. Cap. Class 33, 53–73. doi: 10.1177/030981680909700104

[ref25] Dale J. Kyle D. (2016). Smart humanitarianism: re-imagining human rights in the age of Enterprise. Crit. Sociol. 42, 783–797. doi: 10.1177/0896920516640041

[ref26] Daubs M. S. (2016). The social news network: the appropriation of community labour in CNN’s iReport.

[ref9001] De Gregorio G. (2022). Digital Constitutionalism in Europe: reframing rights and powers in the algorithmic society (1st ed.). Cambridge University Press. doi: 10.1017/9781009071215

[ref27] De Gregorio G. Radu R. (2022). Digital constitutionalism in the new era of internet governance. Int. J. Law Inf. Technol. 30, 68–87. doi: 10.1093/ijlit/eaac004

[ref28] Della Porta D. Chesta R. E. Cini L. (2022). Labour conflicts in the digital age: A comparative perspective. 1st Edn. Bristol: Bristol University Press.

[ref29] Dertouzos M. L. Moses J. (1979). The computer age: A twenty-year view. London: MIT Press.

[ref30] Deshaies C. (2019). *The rise and decline of the cooperative commonwealth Federation in Ontario and Quebec during world war II, 1939—1945* [electronic theses and dissertations], The University of Maine]. Available at: https://digitalcommons.library.umaine.edu/etd/3129/

[ref31] Diani M. (1992). The concept of social movement. Sociol. Rev. 40, 1–25. doi: 10.1111/j.1467-954X.1992.tb02943.x

[ref32] Diani M. (2000). Social movement networks virtual and real. Inf. Commun. Soc. 3, 386–401. doi: 10.1080/13691180051033333

[ref33] Dobusch L. Quack S. (2008). Epistemic communities and social movements: transnational dynamics in the case of creative commons. Cologne: Max Planck Institute for the Study of Societies.

[ref34] Dubeux A. (2013). “Technological incubators of solidarity economy initiatives: a methodology for promoting social innovation in Brazil: collective action, social learning and transdisciplinary research” in The international handbook on social innovation. eds. Moulaert F. MacCallum D. Mehmood A. Hamdouch A. (Cheltenham: Edward Elgar Publishing).

[ref35] Dulong de Rosnay M. Stalder F. (2020). Digital commons. *Internet*. Pol. Rev. 9:1530. doi: 10.14763/2020.4.1530

[ref36] Earl J. Kimport K. (2011). Digitally enabled social change: Activism in the internet age. Cambridge, MA: MIT Press.

[ref37] Eisenstadt T. A. LeVan A. C. Maboudi T. (2017). Constituents before assembly: participation, deliberation and representation in the crafting of new constitutions. 1st Edn. Cambridge, MA: Cambridge University Press.

[ref38] Eyerman R. Jamison A. (2007). Social movements: A cognitive approach (reprinted). Cambridge: Polity Press.

[ref39] Falanga R. (2024). Democratic innovations: is the local scale (still) the ideal laboratory for democracy? Local Gov. Stud. 50, 1052–1061. doi: 10.1080/03003930.2024.2407010, PMID: 40360821

[ref40] Federici S. Linebaugh P. (2019). Re-enchanting the world: Feminism and the politics of the commons. Binghamton, NY: PM Press.

[ref41] Flacks R. (1988). Making history: The American left and the American mind. New York, NY: Columbia University Press.

[ref42] Flanagan K. (2022). *Commoning the City* [electronic theses and dissertations, National University of Ireland Maynooth]. Available at: https://mural.maynoothuniversity.ie/id/eprint/16565/

[ref43] Flesher Fominaya C. (2010). Collective identity in social movements: central concepts and debates: collective identity in social movements. Sociol. Compass 4, 393–404. doi: 10.1111/j.1751-9020.2010.00287.x

[ref44] Flesher Fominaya C. Gillan K. (2017). Navigating the technology-media-movements complex. Soc. Mov. Stud. 16, 383–402. doi: 10.1080/14742837.2017.1338943

[ref45] Foxworth R. Ellenwood C. (2023). Indigenous peoples and third sector research: indigenous data sovereignty as a framework to improve research practices. Volunt. Int. J. Volunt. Nonprofit Org. 34, 100–107. doi: 10.1007/s11266-022-00458-7

[ref46] Fraisse L. (2013). “The social and solidarity-based economy as a new field of public action: a policy and method for promoting social innovation: collective action, social learning and transdisciplinary research” in The international handbook on social innovation. eds. Moulaert F. MacCallum D. Mehmood A. Hamdouch A. (Cheltenham: Edward Elgar Publishing).

[ref47] Fung A. Wright E. O. (2001). Deepening democracy: innovations in empowered participatory governance. Polit. Soc. 29, 5–41. doi: 10.1177/0032329201029001002

[ref48] Future of Life Institute (2023). *Pause Giant AI Experiments: An Open Letter* [Open Letter]. Campbell, CA: Future of Life Institute.

[ref49] Gibson J. J. (1986). The ecological approach to visual perception. Mahwah, NJ: Lawrence Erlbaum Associates, Inc.

[ref50] Giotitsas C. Pazaitis A. Kostakis V. (2015). A peer-to-peer approach to energy production. Technol. Soc. 42, 28–38. doi: 10.1016/j.techsoc.2015.02.002

[ref51] Giraud E. (2014). Has radical participatory online media really ‘failed’? Indymedia and its legacies. Convergence Int. J. Res. New Media Technol. 20, 419–437. doi: 10.1177/1354856514541352

[ref52] Gordon Nembhard J. (2021). “Building a cooperative solidarity commonwealth” in The new systems reader: Alternatives to a failed economy. eds. Speth J. G. Courrier K. (London: Routledge), 273–284.

[ref53] Graham M. Woodcock J. Heeks R. Mungai P. Van Belle J.-P. Du Toit D.. (2020). The Fairwork foundation: strategies for improving platform work in a global context. Geoforum 112, 100–103. doi: 10.1016/j.geoforum.2020.01.023

[ref54] Gray E. G. (2016). *Tom Paine’s iron bridge: Building a United States* (first edition). New York, NY: W.W. Norton & Company.

[ref55] Gray C. (2023). More than extraction: rethinking Data’s colonial political economy. Int. Political Sociol. 17:olad007. doi: 10.1093/ips/olad007

[ref56] Grossman R. L. Adams F. T. (1993). Taking care of business: Citizenship and the charter of incorporation. New Solutions J. Environ. Occup. Health Policy 3, 7–18. doi: 10.2190/NS3.3.c 22913952

[ref57] Guinan J. O’Neill M. (2020). The case for community wealth building. London: Polity.

[ref9002] Gurumurthy A. Chami N. (2020). The intelligent corporation: data and the digital economy (State of Power 2020). Transnational Institute. Available at: https://longreads.tni.org/stateofpower/the-intelligent-corporation-data-and-the-digital-economy

[ref58] Haar J. (2023). Closing the digital divide. Wahba Institute for Strategic Competition - Insight and Analysis. Available online at: https://www.wilsoncenter.org/article/closing-digital-divide (Accessed January 21, 2024).

[ref59] Han E. Levendowski A. Perlin J. (2023). Disrupting data cartels by editing Wikipedia. Yale J. Law Technol. 25, 124–145. doi: 10.2139/ssrn.4390065

[ref60] Hanna T. Lawrence M. Nils P. (2020). A common platform. Common Wealth and Democracy Collaborative. Available online at: https://thenextsystem.org/learn/stories/common-platform-reimagining-data-and-platforms (Accessed January 21, 2024).

[ref61] Hansen J. Pang Y.-S. (2023). Opening industry data: the private sector’s role in addressing societal challenges. Data Policy 5:e19. doi: 10.1017/dap.2023.15

[ref62] Hardt M. Negri A. (2011). Commonwealth (first Harvard University press paperback edition). Cambridge, MA: Belknap Press of Harvard University Press.

[ref63] Hastings A. G. (2024). Strategic roadmap and agenda [strategic plan]. Burlington, MA: Open Mobility Foundation.

[ref64] Hess C. Ostrom E. (2006). Understanding knowledge as a commons: From theory to practice. Cambridge, MA: The MIT Press.

[ref65] Hind D. (2019). *The British digital cooperative: A new model public sector institution* (democracy and governance). The Next System Project, Common Wealth. Available online at: https://thenextsystem.org/bdc#infographic-what-is-the-british-digital-cooperative (Accessed December 10, 2024).

[ref66] Jung M. (2022). Digital capitalism is a mine not a cloud. Transnational Institute, *State of Power 2023* (Digital Power). Available online at: https://www.tni.org/files/2023-04/Digital%20capitalism%20is%20a%20mine%20not%20a%20cloud.pdf (Accessed December 10, 2024).

[ref67] Kemp S. (2023). Digital 2023: global overview report. Data Reportal. Available online at: https://datareportal.com/reports/digital-2023-global-overview-report (Accessed January 21, 2024).

[ref68] Kioupkiolis A. (2022). Digital commons, the political and social change: towards an integrated strategy of counter-hegemony furthering the commons. Ephemera 22, 51–82.

[ref69] Kostakis V. Niaros V. Dafermos G. Bauwens M. (2015). Design global, manufacture local: exploring the contours of an emerging productive model. Futures 73, 126–135. doi: 10.1016/j.futures.2015.09.001

[ref70] Kostakis V. Niaros V. Giotitsas C. (2023). Beyond global versus local: illuminating a cosmolocal framework for convivial technology development. Sustain. Sci. 18, 2309–2322. doi: 10.1007/s11625-023-01378-1

[ref71] Kukutai T. Taylor J. (2016). Indigenous data sovereignty: Toward an agenda. Canberra, ACT: ANU Press.

[ref72] La Follette R. M. (1897). Speeches of Robert M. La Follette—"the danger threatening representative government". Madison, WI: Wisconsin Historical Society.

[ref73] Lanzi D. (2023). Commonism and capabilities. Ephemera. 23, 191–209.

[ref74] Leonardi P. M. (2012). “Materiality, Sociomateriality, and socio-technical systems: what do these terms mean? How are they different? Do we need them?” in Materiality and organizing. eds. Leonardi P. M. Nardi B. A. Kallinikos J.. 1st ed (Oxford: Oxford University Press), 24–48.

[ref75] Leonardi P. M. (2011). When flexible routines meet flexible technologies: affordance, constraint, and the imbrication of human and material agencies. MIS Q. 35:147. doi: 10.2307/23043493

[ref76] Licklider J. (1963). Topics for discussion at the forthcoming meeting of the members and affiliates of the intergalactic computer network. Available online at: http://www.chick.net/wizards/memo.html

[ref77] Linebaugh P. (2008). The magna carta manifesto: Liberties and commons for all. Berkeley, CA: University of California Press.

[ref79] Manski B. (2018). Constituents before Assembly: Participation, Deliberation, and Representation in the Crafting of New Constitutions. By Todd A. Eisenstadt, A. Carl LeVan, and Tofigh Maboudi. New York: Cambridge University Press, 2017. Law & Society Review. 52, 1111–1116. doi: 10.1111/lasr.12376

[ref80] Manski B. (2019). “Methodological approaches to movement waves and the making of history” in The Palgrave handbook of social movements, revolution, and social transformation. ed. Berberoglu B. (Cham: Springer International Publishing), 35–63.

[ref81] Manski B. (2020). Popular Constitutionalism, Powers of Structure, and Strategies of Movement in the Pursuit of Democracy. UC Santa Barbara. ProQuest ID: MANSKI_ucsb_0035D_14892. Merritt ID: ark:/13030/m56x4qxv. Available online at: https://escholarship.org/uc/item/7ch3h41k (Accessed March 5, 2025).

[ref82] Manski S. Manski B. (2018). No gods, no masters, no coders? The future of sovereignty in a Blockchain world. Law Critique 29, 151–162. doi: 10.1007/s10978-018-9225-z

[ref84] Marx K. (2024). Capital: Critique of political economy, Volume 1. Princeton, NJ: Princeton University Press.

[ref85] Mattoni A. (2013). “Technology and social movements” in The Wiley-Blackwell encyclopedia of social and political movements. eds. Snow D. A. Della Porta D. Klandermans B. McAdam D. (Hoboken, NJ: Blackwell Publishing Ltd.).

[ref86] McAdam D. Tarrow S. Tilly C. (2001). Dynamics of contention. 1st Edn. Cambridge: Cambridge University Press.

[ref87] Melucci A. (1998). Inner time and social time in a world of uncertainty. Time Soc. 7, 179–191. doi: 10.1177/0961463X98007002001, PMID: 40372777

[ref88] Milan S. (2016). “Liberated technology: inside emancipatory communication activism” in Civic media. eds. Gordon E. Mihailidis P. (London: The MIT Press), 107–124.

[ref89] Miliband R. (1954). The politics of Robert Owen. J. Hist. Ideas 15:233. doi: 10.2307/2707769

[ref90] Monge F. Barns S. Kattel R. Bria F. (2022). A new data deal: The case of Barcelona. Available online at: https://www.ucl.ac.uk/bartlett/public-purpose/sites/bartlett_public_purpose/files/new_data_deal_barcelona_fernando_barns_kattel_and_bria.pdf (Accessed January 21, 2024).

[ref91] Monsees L. Liebetrau T. Austin J. L. Leander A. Srivastava S. (2023). Transversal politics of big tech. Int. Political Sociol. 17:olac020. doi: 10.1093/ips/olac020

[ref92] Morozov E. (2023). The True Threat of Artificial Intelligence. *New York Times*. Available online at: https://www.nytimes.com/2023/06/30/opinion/artificial-intelligence-danger.html?smid=nytcore-android-share (accessed June 30, 2023).

[ref93] Morris D. (1992). The Mondragon system: cooperation at work. Washington, DC: Institute for Local Self-Reliance.

[ref95] Norman D. A. (2001). The design of everyday things. London: MIT Press.

[ref96] O’Neil M. Pentzold C. Toupin S. (2021). The handbook of peer production. Hoboken, NJ: Wiley Blackwell.

[ref9004] Orlikowski W. J. (2007). Sociomaterial Practices: Exploring Technology at Work. Organ. Stud. 28, 1435–1448. doi: 10.1177/0170840607081138

[ref98] Papadimitropoulos V. Malamidis H. (2024). The transformative potential of platform Cooperativism: the case of CoopCycle. tripleC Inf. Soc. 22, 1–24. doi: 10.31269/triplec.v22i1.1418

[ref99] Pazaitis A. Kostakis V. Drechsler W. (2022). Towards a theory of value as a commons. Int. J. Commons 16, 248–262. doi: 10.5334/ijc.1153

[ref100] Pelkey J. L. Russell A. L. Robbins L. G. (2022). Circuits, packets, and protocols: Entrepreneurs and computer communications, 1968–1988. First Edn. New York, NY: Association for Computing Machinery.

[ref101] Pentland A. Lipton A. Hardjono T. (2021). Building the new economy: Data as capital. London: The MIT Press.

[ref102] Platform Cooperativism Consortium and IT for Change. (2023b). The Thiruvananthapuram declaration on a new innovation ecosystem for our collective digital futures. In Roots of resilience conference. Platform Cooperativism Consortium. Available online at: https://platform.coop/blog/the-thiruvananthapuram-declaration-on-a-new-innovation-ecosystem-for-our-collective-digital-futures-2/ (Accessed February 24, 2024).

[ref103] Platform Cooperativism Consortium and IT for Change. (2023a). Roots of Resilience—Conference (2023). In Roots of Resilience Conference. Platform Cooperativism Consortium. Available online at: https://platform.coop/events/roots-of-resilience/

[ref104] Podmore F. (1905). Robert Owen and Cooperation. Econ. J. 15:257. doi: 10.2307/2221015

[ref9006] R A A. Sulfath J. Kazi S. S. (2022). Advancing data justice research and practice (Country Report - India). Digital empowerment foundation. Available at: https://advancingdatajustice.org/wp-content/uploads/2022/04/Advancing-Data-Justice-Research-and-Practice-India-Report—Digital-Empowerment-Foundation.pdf

[ref106] Reich R. Sahami M. Weinstein J. M. (2021). *System error: where big tech went wrong and how we can reboot* (first edition). New York, NY: Harper Collins Publishers.

[ref107] Rene G. Mapes D. (2019). The spatial web: How web 3.0 will connect humans, machines and AI to transform the world. Los Angeles, CA: Gabriel René and Dan Mapes.

[ref108] Restakis J. (2021). “Cooperative commonwealth and the partner state” in The new systems reader: Alternatives to a failed economy. eds. Speth J. G. Courrier K. (London: Routledge), 362–384.

[ref109] Ridley-Duff R. Bull M. (2021). Common pool resource institutions: the rise of internet platforms in the social solidarity economy. Bus. Strateg. Environ. 30, 1436–1453. doi: 10.1002/bse.2707

[ref110] Robey D. Raymond B. Anderson C. (2012). “Theorizing information technology as a material artifact in information systems research” in Materiality and organizing. eds. Leonardi P. M. Nardi B. A. Kallinikos J.. 1st ed (Oxford: Oxford University Press), 216–236.

[ref111] Rozas D. Tenorio-Fornés A. Hassan S. (2021). Analysis of the potentials of Blockchain for the governance of global digital commons. Front. Blockchain 4:577680. doi: 10.3389/fbloc.2021.577680

[ref112] Saner R. Yiu L. Nguyen M. (2018). “Platform cooperatives: the social and solidarity economy and the future of work,” in *UN inter-Agency Task Force on Social and Solidarity Economy*. Geneva: UNTFSSE International Conference.

[ref113] Scholz R. T. (2023). Own this! How platform cooperatives help workers build a democratic Internet. London: Verso.

[ref114] Scholz T. Schneider N. (2017). Ours to hack and to own: The rise of platform cooperativism, a new vision for the future of work and a fairer internet. New York City, NY: OR Books.

[ref9003] Sekalala S. Dagron S. Forman L. Meier B. M. (2020). Analyzing the human rights impact of increased digital public health surveillance during the COVID-19 crisis. Health Hum. Rights, 22, 7–20. PMC7762901 33390688

[ref115] Singh P. J. Vipra J. (2019). Economic rights over data: a framework for community data ownership. Development 62, 53–57. doi: 10.1057/s41301-019-00212-5, PMID: 39310270

[ref116] Smith G. (2024). We need to talk about climate: How citizens’ assemblies can help us solve the climate crisis. London: University of Westminster Press.

[ref117] Spatial Web Foundation (2023). The spatial web charter. Piscataway, NJ: Spatial Web Foundation.

[ref118] Stockwell N. Manski B. (2020). Indymedia and media activism at the turn of the millennium. Social. Democr. 34, 216–227. doi: 10.1080/08854300.2019.1841523

[ref9005] Tarrow S. G. (2011). Power in Movement: Social Movements and Contentious Politics (3rd ed.). Cambridge University Press. doi: 10.1017/CBO9780511973529

[ref119] Taylor V. (2000). Mobilizing for change in a social movement society. Contemp. Sociol. 29:219. doi: 10.2307/2654946

[ref120] Taylor A. (2014). The people’s platform: Taking back power and culture in the digital age. New York, NY: Metropolitan Books.

[ref121] Teubner G. (2010). “Fragmented foundations: societal constitutionalism beyond the nation state” in The twilight of constitutionalism? eds. Dobner P. Loughlin M.. 1st ed (Oxford: Oxford University Press), 327–342.

[ref122] Teubner G. Golia A. Jr Societal Constitutionalism in the Digital World: An Introduction (2023). Max Planck Institute for Comparative Public Law & International Law (MPIL) Research Paper No. 2023-11, Forthcoming in: Angelo Jr Golia and Gunther Teubner (eds.), Digital Constitution: On the Transformative Potential of Societal Constitutionalism. Symposium: Indiana Journal of Global Legal Studies. 30, 1–24. Available at SSRN: https://ssrn.com/abstract=4433988

[ref123] Tilly C. Wood L. (2020). Social movements as politics. 4th Edn. London: Routledge, 3–17.

[ref124] Torvalds L. Diamond D. (2002). Just for fun: The story of an accidental revolutionary. 1st Edn. New York, NY: Harper.

[ref125] Touraine A. (1981). The voice and the eye: an analysis of social movements. Cambridge, MA: Cambridge University Press.

[ref126] Touraine A. (1983). Solidarity: The analysis of a social movement: Poland, 1980–1981. Cambridge, MA: Cambridge University Press.

[ref127] Touraine A. (1988). Return of the actor: Social theory in postindustrial society. Minnesota, MN: University of Minnesota Press.

[ref128] Treré E. Jeppesen S. Mattoni A. (2017). Comparing digital protest media imaginaries: anti-austerity movements in Greece, Italy & Spain. tripleC Inf. Soc. 15, 404–422. doi: 10.31269/triplec.v15i2.772

[ref129] Viljoen S. (2021). A relational theory of data governance. Yale Law J. 131, 573–654. Available online at: https://www.yalelawjournal.org/pdf/131.2_Viljoen_1n12myx5.pdf

[ref130] Walter M. Kukutai T. Russo Carroll S. Rodriguez-Lonebear D. (2021). Indigenous data sovereignty and policy. London: Routledge.

[ref131] Winch D. M. (1977). Political economy and the economic polity. Can. J. Econ. 10:547. doi: 10.2307/134290

[ref132] Winner L. (1980). Do artifacts have politics? Daedalus 109, 121–136.

[ref133] Wolfson T. (2014). Activist laboratories of the 1990’s: the roots of technological determinism in contemporary social movements. Cult. Stud. 28, 657–675. doi: 10.1080/09502386.2014.888931

[ref134] World Economic Forum (2024). *The digital economy* (version 2023) [encyclopedia entry]. Cologny: World Economic Forum.

[ref135] Wright E. O. (2010). Envisioning real utopias. London: Verso.

[ref136] Young A. F. (2006). Liberty tree: Ordinary people and the American revolution. New York, NY: New York University Press.

[ref137] Zarevich E. (2022). Marie curie and polish resistance. JSTOR Daily. Available online at: https://daily.jstor.org/marie-curie-and-polish-resistance/

[ref138] Zuberi T. Bonilla-Silva E. (2008). White logic, white methods: Racism and methodology. Lanham, MD: Rowman & Littlefield Publishers.

[ref139] Zuboff S. (2019). ““We make them dance”: surveillance capitalism, the rise of Instrumentarian power, and the threat to human rights” in Human rights in the age of platforms. ed. Jørgensen R. F. (New York, NY: The MIT Press), 3–52.

